# Commercial Hospitals in the West

**Published:** 1840

**Authors:** 


### COMMERCIAL HOSPITALS IN THE WEST.

We are glad to find this subject revived in Congress, after a sleep of more than two years. It now comes up in the form of a letter from Dr. Lawson, Surgeon General, U. S. A., to the Secretary of War, which letter was lately transmitted to the Senate, by the President, in a special message.

It seems that in 1837, a commission of three army surgeons, of which Dr. Harney was president, made a selection of seven sites; but where does not appear in the documents before us. Land was purchased at each place, and deeds, in the nature of escrols, were executed to the government; but no money paid, and the bargains were liable to be annulled by said Government. It has not, however seen fit, either to confirm or annul, and there the matter rests.

In the clangor of partisan warfare, it is not always easy for the voice of suffering humanity to be heard; however, we do not despair. True it is, the President has *not recommended* the appropriation necessary to these purchases, but the Secretary and Surgeon General have done *their* duty, and we may hope for the best. Let the latter speak for himself:

"The claims of those untiring and intrepid sons of the West, who from the heads of the Alleghany and Monongahela, and from the banks of the Ohio and other concurrent streams, voyage it to the lowest point of navigation in our western region, and of those hardy and equally adventurous spirits, who from the upper Mississippi and Missouri, sweep the western waters to the very verge of the ocean, cannot be resisted. Sooner or later the cries of their suffering comrades, who in braving the dangers of fire and water, of clime and country, for the general welfare, are frequently stricken down by disease and accident, will be heard and must be heard. Let us, then, in obedience to the dictates of humanity and of common justice, call upon Congress to extend, at once, a helping hand to this much to be admired, yet little cherished band of navigators; nay, to dispense to them, and with a liberal hand, the willing offerings of a nation's gratitude."

We earnestly commend this subject to our brethren in the West, as one every way entitled to their consideration. D.

